# The Impact of SLC2A8 RNA Interference on Glucose Uptake and the Transcriptome of Human Trophoblast Cells

**DOI:** 10.3390/cells13050391

**Published:** 2024-02-24

**Authors:** Aleksandra Lipka, Łukasz Paukszto, Victoria C. Kennedy, Amelia R. Tanner, Marta Majewska, Russell V. Anthony

**Affiliations:** 1 Department of Gynecology and Obstetrics, School of Medicine, Collegium Medicum, University of Warmia and Mazury in Olsztyn, 10-045 Olsztyn, Poland; 2 Department of Botany and Nature Protection, Faculty of Biology and Biotechnology, University of Warmia and Mazury in Olsztyn, 10-727 Olsztyn, Poland; pauk24@gmail.com; 3 College of Veterinary Medicine, Colorado State University, Fort Collins, CO 80523, USA; tori.kennedy@colostate.edu (V.C.K.); amelia.tanner@cuanschutz.edu (A.R.T.); 4 Department of Human Physiology and Pathophysiology, School of Medicine, University of Warmia and Mazury in Olsztyn, 10-082 Olsztyn, Poland; marta.majewska@uwm.edu.pl

**Keywords:** SLC2A8, GLUT8, trophoblast, placenta, pregnancy, ACH-3P, RNA interference, RNAseq, glucose uptake

## Abstract

While glucose is the primary fuel for fetal growth, the placenta utilizes the majority of glucose taken up from the maternal circulation. Of the facilitative glucose transporters in the placenta, SLC2A8 (GLUT8) is thought to primarily function as an intracellular glucose transporter; however, its function in trophoblast cells has not been determined. To gain insight into the function of SLC2A8 in the placenta, lentiviral-mediated RNA interference (RNAi) was performed in the human first-trimester trophoblast cell line ACH-3P. Non-targeting sequence controls (NTS RNAi; *n* = 4) and *SLC2A8* RNAi (*n* = 4) infected ACH-3P cells were compared. A 79% reduction in *SLC2A8* mRNA concentration was associated with an 11% reduction (*p* ≤ 0.05) in ACH-3P glucose uptake. NTS RNAi and *SLC2A8* RNAi ACH-3P mRNA were subjected to RNAseq, identifying 1525 transcripts that were differentially expressed (|log2FC| > 1 and adjusted *p*-value < 0.05), with 273 transcripts derived from protein-coding genes, and the change in 10 of these mRNAs was validated by real-time qPCR. Additionally, there were 147 differentially expressed long non-coding RNAs. Functional analyses revealed differentially expressed genes involved in various metabolic pathways associated with cellular respiration, oxidative phosphorylation, and ATP synthesis. Collectively, these data indicate that *SLC2A8* deficiency may impact placental uptake of glucose, but that its likely primary function in trophoblast cells is to support cellular respiration. Since the placenta oxidizes the majority of the glucose it takes up to support its own metabolic needs, impairment of SLC2A8 function could set the stage for functional placental insufficiency.

## 1. Introduction

The successful outcome of pregnancy requires the functional integration of three compartments: the maternal, placental, and fetal compartments. This is especially true when one considers the umbilical uptake of nutrients in support of fetal growth. Glucose, the primary energy source for fetal and placental oxidative processes [1], is entirely derived from the maternal circulation throughout most of gestation, as fetal gluconeogenesis does not begin until the very near term [2,3,4,5]. Glucose is transferred across the placenta, from maternal to fetal circulation, down a maternal–fetal glucose concentration gradient via facilitative diffusion [6]. Beyond the maternal–fetal glucose concentration gradient, placental metabolism, blood flow, and the availability and activity of specific glucose transporters can impact the efficiency of placental glucose transport [1,7].

Fourteen different facilitative glucose transporters (GLUTs) have been identified, which differ in terms of substrate specificity, kinetics, distribution, location, and regulatory mechanisms [8]. Within the human placenta, GLUT1 (SLC2A1), GLUT3 (SLC2A3), GLUT4 (SLC2A4), GLUT8 (SLC2A8), GLUT9 (SLC2A9), GLUT10 (SLC2A10), and GLUT12 (SLC2A12) have been detected [6,9]. Due to its abundance and distribution, SLC2A1 is considered the primary glucose transporter within the placenta [10,11], but is supported by SLC2A3, which has a higher affinity and transport capacity [12], at least during the first half of gestation [13,14] or in some cases of intrauterine growth restriction [15].

The maternal to fetal glucose gradient, which drives facilitated diffusion, results in part from the extensive oxidation of glucose by the placenta. The placenta is a highly metabolic organ, utilizing 80 and 72% of the glucose taken up by the uterus at mid- and late-gestation, respectively [16,17]. In contrast to SLC2A1 and SLC2A3, which are involved in glucose uptake and transfer to the fetus, SLC2A8 (GLUT8) is a class III glucose transporter, primarily localized in endosomes, lysosomes, and endoplasmic reticulum membranes [18], and is thought to catalyze hexose transport across intracellular membranes. However, in the mouse blastocyst, in response to insulin, SLC2A8 localization shifts to the plasma membrane, and is believed to be responsible for insulin-stimulated glucose uptake by the blastocyst [19,20]. *Slc2A8^−/−^* mice were viable and developed normally [21,22], but did exhibit alterations in the brain, heart, and sperm cells. The reduction in sperm cell motility with *Slc2A8^−/−^* males appears to be associated with reduced mitochondrial membrane potential and ATP availability in sperm [22], which fits with the role of SLC2A8 as an intracellular transporter. While these results do not support an important role for SLC2A8 in placental glucose transport, culturing murine embryos with *Slc2A8* antisense RNA resulted in increased blastocyst apoptosis and reduced viability [20], and Limesand et al. [23] reported diminished placental expression of *SLC2A8* in a sheep model of placental insufficiency resulting in intrauterine growth restriction (IUGR). As the function of SLC2A8 in placental glucose uptake and metabolism has not been fully interrogated, we hypothesized that reduction in SLC2A8 in human trophoblast cells could reduce glucose uptake and alter metabolic processes important in glucose oxidation. Therefore, our objective was to use lentiviral-mediated RNA interference (RNAi) to determine the impact of *SLC2A8* deficiency on the trophoblast transcriptome and glucose uptake.

## 2. Materials and Methods

All procedures were approved by the Colorado State University Institutional Biosafety Committee (17-039B).

### 2.1. Lentivirus Vector Construction and Virus Generation

To target *SLC2A8* mRNA for RNA interference, four shRNAs homologous to human *SLC2A8* (Table 1) were initially inserted into pLKO.1 (plasmid #10878; Addgene, Cambridge, MA, USA). The human U6 promoter and *SLC2A8* shRNA were then shuttled into pLentiLox3.7 (pLL3.7 plasmid #11795; Addgene, Cambridge, MA, USA), replacing the mouse U6 promoter, as extensively described in Baker et al. [24]. All constructs were subjected to capillary sequencing (QuintaraBio, Bay Area, CA, USA) to verify authenticity. The non-targeting sequence (NTS) control LL3.7 plasmid was previously described in Jeckel et al. [25].

The lentivirus was generated as described previously [24,26,27]. Briefly, 293FT (Thermo Fisher Scientific, Waltham, MA, USA) cells were grown to 70–80% confluence in a 15 cm tissue culture plate in high-glucose DMEM supplemented with 10% *v*/*v* fetal bovine serum (FBS; Peak Serum, Bradenton, FL, USA) and 1% Penicillin/Streptomycin/Amphotericin (PSA; Corning Life Sciences, New York, NY, USA) at 37 °C and 5% CO_2_. To generate second-generation lentiviral particles, pLL3.7-NTS, and pLL3.7-SLC2A8 plasmids, psPAX2 packaging plasmid (Addgene, Cambridge, MA, USA), and pMD2.G envelope plasmid (Addgene, Cambridge, MA, USA) were used. For each 15 cm plate, the transfection mix was prepared as follows: 8.82 μg of LL3.7 (NTS- or SLC2A8-shRNA), 6.66 μg of psPAX2, and 2.70 μg of pMD2.G were mixed with 180 μL of polyfect transfection reagent (Qiagen, Hilden, Germany), and the final volume was brought up to 675 μL using serum and antibiotic-free DMEM media. The transfection mixture was added to 293FT cells along with 15 mL of complete medium. After 24 h of incubation in the transfection reagent, the medium was aspirated, and fresh complete DMEM medium was added. Seventy-two hours after transfection, cell culture supernatants were collected and ultracentrifuged over a 20% *w*/*v* sucrose cushion at 47,000× *g* for 2 h at 4 °C. After ultracentrifugation, lentiviral pellets were resuspended in 1xPBS and stored in aliquots at −80 °C. Aliquots of the lentivirus were titered as described previously [24].

### 2.2. In Vitro SLC2A8 RNAi of Human Trophoblast Cells

The human first-trimester trophoblast cell line ACH-3P was used for this study to target *SLC2A8* mRNA by RNAi. ACH-3P are hybrid cells generated by the fusion of primary first-trimester human trophoblast cells (12 weeks of gestation) with AC1-1 cells, a human choriocarcinoma cell line [28]. ACH-3P cells were cultured in Ham’s F-12 medium (Gibco, Thermo Fisher Scientific, Waltham, MA, USA) supplemented with 10% *v*/*v* FBS and 1% PSA at 37 °C and 5% CO_2_.

ACH-3P cells were infected with either LL3.7-NTS (NTS RNAi) or the four LL3.7-SLC2A8 lentiviruses at a multiplicity of infection (MOI) of 100 and 500 transducing units per cell in a 96-well plate. Transfected cells were expanded until near-confluence was obtained in a 15 cm tissue culture plate. The concentration of *SLC2A8* mRNA in transfected ACH-3P cells was assessed by quantitative real-time reverse transcriptase PCR (qPCR).

### 2.3. RNA Extraction and qPCR

Total RNA was isolated from cell pellets using a RNeasy Kit (Qiagen, Hilden, Germany) according to the manufacturer’s protocol. RNA quality and concentration, measured by the 260/280 nm absorbance ratio, were assessed using a plate reader (BioTek, Winooski, VT, USA), and samples were stored at −80 °C until use. Complementary DNA was generated from 1 μg of total RNA using an iScript™ Reverse Transcription Supermix (BioRad, Hercules, CA, USA). Quantitative real-time PCR was conducted using iQ™ SYBR^®^ Green Supermix (BioRad, Hercules, CA, USA). The primer sequences used are presented in Table 2. For analysis, a PCR product for each gene was generated and cloned using the StrataClone PCR Cloning Kit (Agilent Technologies, Santa Clara, CA, USA). Each PCR product was sequenced to verify the amplification of the correct mRNA (GeneWIZ, Azenta Life Sciences, Burlington, MA, USA). By amplifying the PCR product from each gene’s plasmid, a standard curve ranging from 1 × 10^2^ to 1 × 10^−6^ pg was generated. The starting quantity (pg) of each mRNA was normalized to the starting quantity of ribosomal protein S15 (*RPS15*). Control (NTS RNAi) and *SLC2A8*-shRNA treatments (*SLC2A8* RNAi) were compared by unpaired Students *t*-test, with *p* < 0.05 considered as statistically significant.

### 2.4. Glucose Uptake Assay

Glucose uptake assay was performed with a commercially available kit (Dojindo, Rockville, MD, USA) according to the manufacturer’s protocol. Briefly, NTS RNAi and *SLC2A8* RNAi ACH-3P cells were seeded in 10 replicates on a 96-well plate (black with clear bottom) with a seeding density of 10,000 cells per well and incubated overnight in 100 µL of regular Ham’s F-12 medium. The next day, the culture medium was removed and cells were washed twice with pre-warmed glucose- and serum-free medium (Gibco, Thermo Fisher Scientific, Waltham, MA, USA). Then, pre-warmed glucose- and serum-free medium was added and the plate was incubated at 37 °C for 15 min in a 5% CO_2_ incubator. Next, the medium was removed and a pre-warmed solution of glucose probe (fluorescent 2-deoxy-d-glucose) was added and incubated for 15 min (37 °C and 5% CO_2_). After incubation and probe removal, cells were washed twice with ice-cold WI solution and then incubated with an ice-cold WI solution at room temperature for 5 min. WI solution was exchanged with ice-cold WI solution and fluorescence was measured on a micro-plate reader (BioTek, Winooski, VT, USA) with excitation and emission wavelengths set to 360/40 and 460/40, respectively.

### 2.5. RNAseq and Data Analysis

Assessment of the RNA samples’ integrity and quality, library generation, and RNA-Seq (2 × 150 cycles, 80 million paired-end reads/sample; NovSeq6000, Illumina, San Diego, CA, USA) was conducted by the Genomics Shared Resource Core Facility, University of Colorado Anschutz Medical Campus (Aurora, CO, USA). The sequencing data from this study have been submitted to the European Nucleotide Archive under accession no. PRJEB71894 (https://www.ebi.ac.uk/ena; accessed on 16 January 2024).

### 2.6. Quality Control and Mapping Processes

The raw paired-end reads quality was assessed using the FastQC software v. 0.11.7 (www.bioinformatics.babraham.ac.uk, accessed on 25 April 2023). Preprocessing with Trimmomatic software v. 0.32 [29] included removal of Illumina adaptors and poly(A) stretches, exclusion of low-quality reads (Phred cutoff score ≤ 20; calculated on both ends of reads and with 10bp frameshift), and trimming of reads to equal length of 90 bp. Then, STAR software v. 2.4 (https://github.com/alexdobin/STAR, accessed on 25 April 2023) was used to map cleaned paired-end reads to the reference soft-masked human genome (Homo_sapiens.GRCh38) with ENSEMBL/GENCODE annotation (Homo_sapiens.GRCh38.105.gtf). Conversion of BAM to SAM format was performed using Samtools v. 1.12 software [30]. Next, to remove multi-mapped reads and retain uniquely aligned reads in the SAM file, the Picard tool was used. StringTie v. 1.3.3 (https://ccb.jhu.edu/software/stringtie, accessed on 28 April 2023) was applied to obtain new annotations by merging an Ensembl GTF file with reads mapped to the reference genome [31]. Count expression values were estimated by ballgown v 2.34.0 software [32] and a prepDE.py Python script (stringtie module). All transcript sequences were extracted to the FASTA file using a gffread script (https://github.com/gpertea/gffread, accessed on 6 May 2023). Memory intensive processes such as mapping, SAM to BAM conversion, and expression level calculation were performed at the Regional IT Center of University of Warmia and Mazury in Olsztyn, Poland.

### 2.7. Transcriptome Profiling of Protein-Coding Genes

Transcriptome profiling was performed as previously described [33,34,35]. Briefly, to obtain the stringent results of differentially expressed transcribed active regions (DE-TARs), the count gene expression matrix was calculated by the ballgown statistical method [32]. The changes in gene expression levels were considered significant when statistical test values (adjusted *p*-value) were lower than 0.05 and logarithmic fold change was lower than −1 or higher than 1. According to GENCODE annotation, DE-TARs were divided into protein-coding genes (DEGs), long non-coding RNAs (DElncRNAs), and other non-coding RNAs (ncRNAs). The research focused mostly on two categories (DEGs and DElncRNAs) and expression values between these transcripts. Candidate DEGs and DElncRNAs were visualized in a volcano and heatmap plots with gplots and circlize Bioconductor R packages (http://www.r-project.org/, accessed on 18 May 2023). The correlation between coding and lncRNA transcripts was calculated using Pearson correlation metric implemented in the rcorr function (Hmisc R package). The obtained DEGs were annotated by the Gene Ontology (GO) and KEGG pathway database using g.Profiler [36] v.0.2.2 software with the g:SCS algorithm (*p* < 0.05).

### 2.8. qPCR Validation of the RNAseq Results

Quantitative PCR was performed, as described earlier, using the same RNA samples as used for RNAseq. Genes for validation were selected among detected DEGs that were assigned to essential GO and KEGG processes. qPCR was performed with predesigned TaqMan assays (Thermo Fisher Scientific, Waltham, MA, USA) for *MT-ND6* (Hs02596879_g1), *MT-CO1* (Hs02596864_g1), *IDH3G* (Hs00188065_m1), *MT-ND5* (Hs02596878_g1), *DLD* (Hs00164401_m1), *CBR4* (Hs00379036_m1), *SURF1* (Hs00894550_m1), *MPC2* (Hs00967250_m1), *NDUFA4L2* (Hs00220041_m1), and *ETFRF1* (Hs01390827_g1), normalized to the level of *RPS15* (Hs01358643_g1) by ΔΔCt, and statistically compared by the unpaired Student’s *t*-test.

## 3. Results

### 3.1. SLC2A8 RNAi in ACH-3P Cells

**Figure 1:**
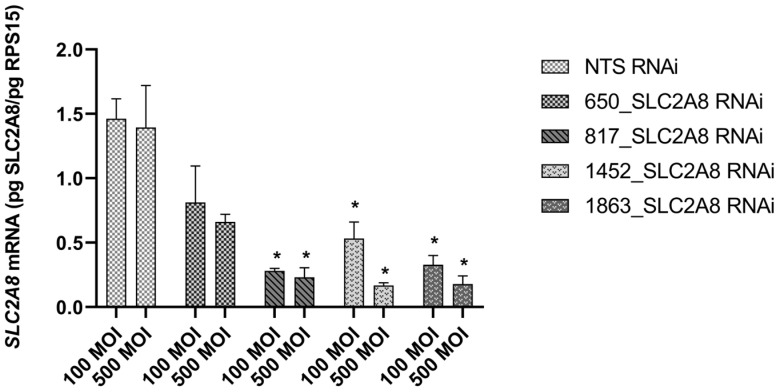
Effect of lentivirus-mediated RNA interference on *SLC2A8* mRNA level in ACH-3P cells. Concentration of *SLC2A8* mRNA was measured by qPCR. Data are shown as mean values ± SEM. * *p* ≤ 0.05 when *SLC2A8*-deficient cell lines are compared with controls (NTS RNAi).

**Figure 2:**
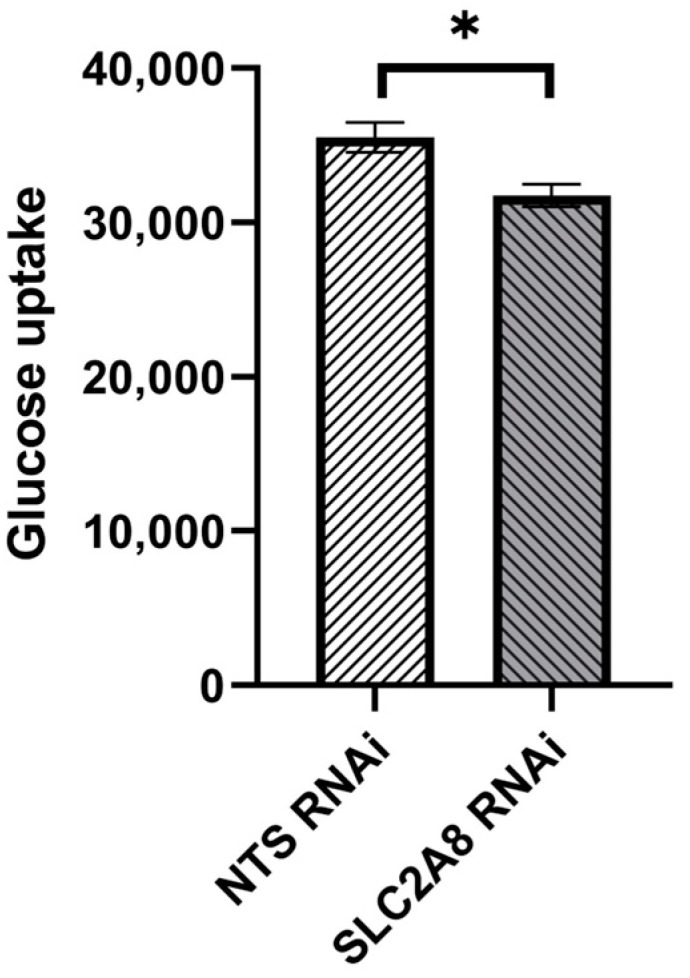
Effect of lentivirus-mediated *SLC2A8* RNAi on glucose uptake in ACH-3P cells. Data are shown as mean values ± SEM. * *p* ≤ 0.05 when *SLC2A8*-deficient cell lines are compared with controls (NTS RNAi).

To target *SLC2A8* mRNA for degradation, lentiviral-mediated RNAi was employed in ACH-3P cells, utilizing four distinct *SLC2A8* shRNAs and a NTS shRNA control. As evidenced in Figure 1, three out of four of the shRNAs resulted in significant depletion of *SLC2A8* mRNA, at both 100 and 500 MOI. The 817 shRNA (Table 1) provided the most consistent RNAi of *SLC2A8*, and was selected for further analysis at 500 MOI, which is now designated as *SLC2A8* RNAi. The glucose uptake by *SLC2A8* RNAi was compared to NTS RNAi ACH-3P cells, and the 79% reduction in *SLC2A8* mRNA (Figure 1) was associated with an 11% decrease in glucose uptake (Figure 2).

### 3.2. RNAseq Statistics

**Figure 3:**
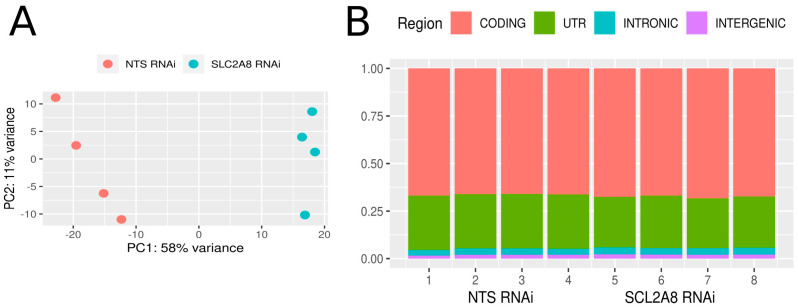
Expression profile overview. (**A**) Graphical representation of the first (PC1) and second (PC2) principal components affecting the sample expression pattern of control (NTS RNAi) and *SLC2A8*-deficient ACH-3P libraries (*SLC2A8* RNAi). (**B**) distribution of reads mapped to the human genome within intergenic, intronic, UTR, and coding regions.

RNAseq analysis was conducted on four replicates of ACH-3P mRNA derived from NTS RNAi and *SLC2A8* RNA cell lines. High-throughput sequencing on the NovaSeq platform (Illumina) generated 949,834,284 raw paired-end reads. After trimming, 96.4% of reads with good quality were uniquely mapped (Table 3) to the reference human genome, and only 3.6% of reads were mapped to multiple loci. The mean percentage distribution of aligned bases was as follows: 2.01% were derived from intergenic regions, 3.36% from intronic regions, 27.75% from untranslated regions, and 66.68% from coding regions (Figure 3). The sequencing revealed 100,246 transcripts that belong to 31,567 transcribed active regions (TARs).

### 3.3. SLC2A8 Deficiency Alters Trophectodermal Gene Expression

**Figure 4:**
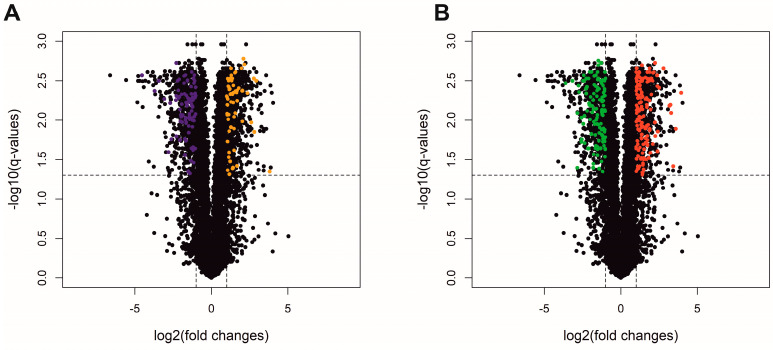
Volcano plot depicts log2FC plotted against log-normalized *p*-values of lncRNA (**A**) and protein-coding genes (**B**) in the deficiency of *SLC2A8* expression. The dotted horizontal line indicates a negative logarithmic adjusted *p* ≤ 0.05 cut-off. Dotted vertical lines indicate cut-off values of logFC. (**A**) Orange dots represent upregulated differentially expressed lncRNA (DElncRNA); purple dots refer to downregulated DElncRNA. (**B**) Red dots illustrate upregulated differentially expressed genes (DEGs); green dots represent downregulated DEGs. Black dots are not significant transcripts, according to the ballgown method (**A**,**B**).

**Figure 5:**
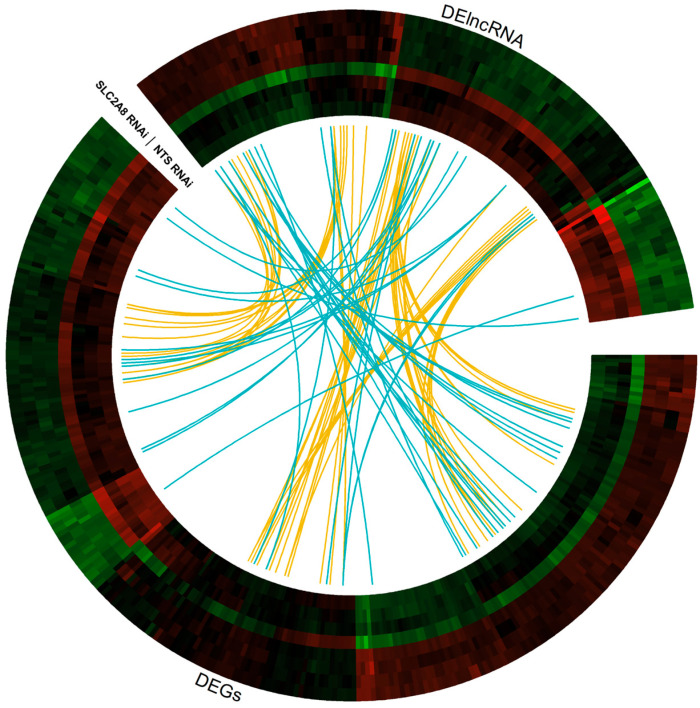
Circular heatmap visualization of differentially expressed genes (DEGs) and differentially expressed lncRNA (DElncRNA) resulting from the deficiency in *SLC2A8* in ACH-3P cells. The eight tracks visualize the normalized (Z-score; red–green scale) expression profiles for DEGs and DElncRNA in each of NTS RNAi and *SLC2A8* RNAi samples. The most inner track shows the correlation links between the co-expressed DEGs and DElncRNA, whereas blue links depict positive and yellow negative (<−0.9) Euclidean correlation > 0.9.

The most stringent DE statistical method, ballgown, revealed 1525 DE-TARs (|log2FC| > 1 and adjusted *p* ≤ 0.05). Among DE-TARs, 273 were classified as DEGs localized in a range of protein-coding genes and 147 were signed as DElncRNAs. The rest of the DE-TARs were other types of non-coding RNAs with unknown or uncertain molecular function. The logarithmic values of fold change (log2FC) and statistical significance are visualized on a Volcano plot (Figure 4). Among differentially expressed genes (DEGs) detected between NTS controls and *SLC2A8*-deficient ACH-3P cells, 148 were upregulated and 125 were downregulated (Figure 5).

**Figure 6:**
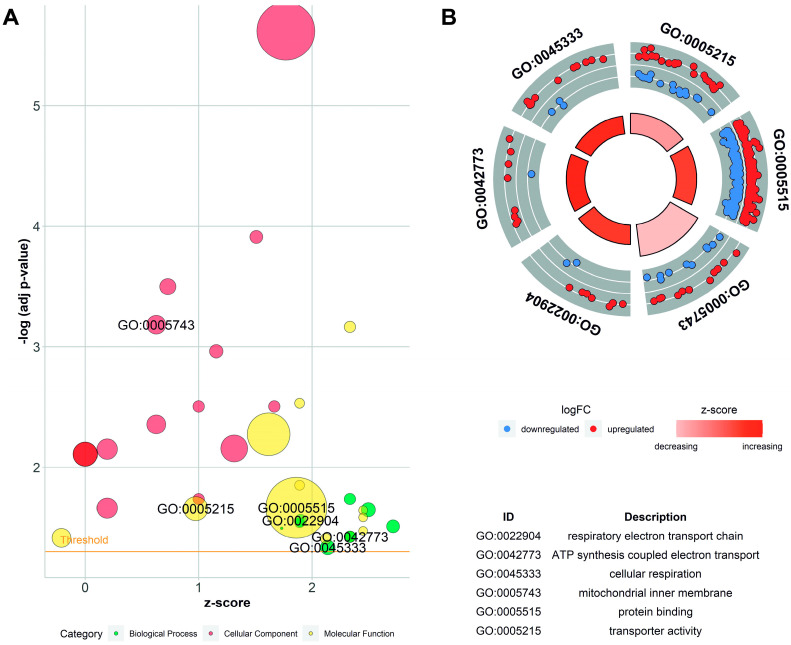
Enrichment ontology visualization. (**A**) Gene Ontology (GO) bubble chart of the assigned ontology terms (biological process—BP, cellular components—CC, and metabolic function—MF). Circle size is proportional to the logarithmic scale of adjusted *p*-value in enrichment GO analysis. Z-score is calculated from the number of up- and downregulated genes enriched in each GO term. (**B**) Circos visualization of selected GO processes related to mitochondria function. Red dots illustrate upregulated differentially expressed genes (DEGs); blue dots represent downregulated DEGs.

Annotations were grouped into 10 biological process (BP), 11 molecular function (MF), and 14 cellular component (CC) categories (Figure 6). Out of the identified DEGs, 327, 326, and 314 coding and non-coding genes were annotated within functional Gene Ontology (GO) database categories BP, CC, and MF, respectively.

**Figure 7:**
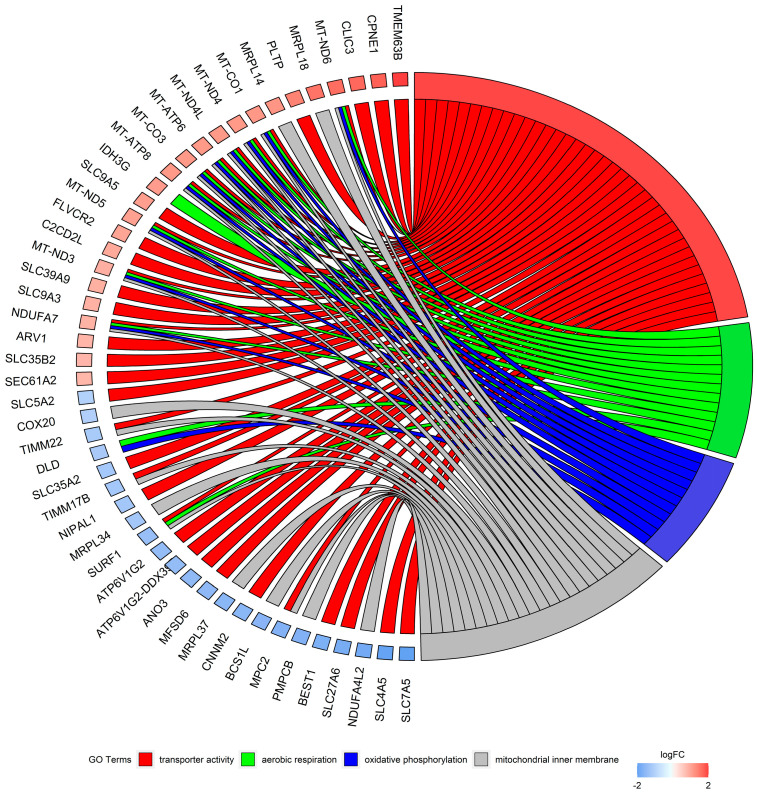
Circos plot represents four significantly enriched Gene Ontology (GO) terms associated with detected differentially expressed genes (DEGs). Gene symbols with logarithmic values (blue-red scale) of fold change (logFC) are located on the left side of the circos. Four color links merge genes with GO annotations.

The DEGs enriched in GO terms were cross-overlapped (Figure 7). The genes involved in the top five BP classes carried out aerobic electron transport chain (9 DEGs out of 87 genes as a count of term size), aerobic respiration (13/192), respiratory electron transport chain (10/115), mitochondrial electron transport, NADH to ubiquinone (7/51), and oxidative phosphorylation (11/142). The top five terms of MF were oxidoreduction-driven active transmembrane transporter activity (9/71); NAD(P)H dehydrogenase (quinone) activity (7/46); catalytic activity (124/5716); oxidoreductase activity, acting on NAD(P)H, quinone or similar compound as acceptor (7/58); and protein binding (260/14,799). In the CC category, the most abundant significantly enriched terms were cytoplasm (233/12,193), respirasome (11/103), mitochondrial protein-containing complex (17/277), mitochondrial inner membrane (23/500), and inner mitochondrial membrane protein complex (12/154). The KEGG pathway enrichment analysis revealed that DEGs were categorized into 10 pathways, including the oxidative phosphorylation pathway. Annotations and functional assignments of the identified DEGs indicate that *SLC2A8* deficiency mostly affects mitochondria functioning.

### 3.4. Validation

**Figure 8:**
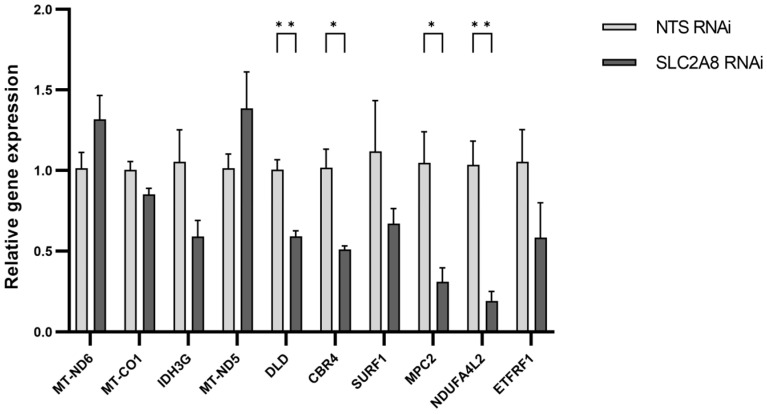
The mRNA expression of selected genes obtained using real-time PCR. The expression values were normalized to *RPS15* housekeeping gene expression. * *p* ≤ 0.05, ** *p* ≤ 0.01 when *SLC2A8*-deficient samples are compared with controls (NTS RNAi). Abbreviations: MT-ND6 (Mitochondrially Encoded NADH:Ubiquinone Oxidoreductase Core Subunit 6); MT-CO1 (Mitochondrially Encoded Cytochrome C Oxidase I); IDH3G (Isocitrate Dehydrogenase (NAD(+)) 3 Non-Catalytic Subunit Gamma); MT-ND5 (Mitochondrially Encoded NADH:Ubiquinone Oxidoreductase Core Subunit 5); DLD (Dihydrolipoamide Dehydrogenase); CBR4 (Carbonyl Reductase 4); SURF1 (SURF1 Cytochrome C Oxidase Assembly Factor); MPC2 (Mitochondrial Pyruvate Carrier 2); NDUFA4L2 (NDUFA4 Mitochondrial Complex Associated Like 2); ETFRF1 (Electron Transfer Flavoprotein Regulatory Factor 1).

The genes selected for qPCR validation were chosen based on the assessment of the function, expression values, and read distribution within libraries. Four over-expressed genes (*MT-ND6*, *MT-CO1*, *IDH3G*, and *MT-ND5*) and six under-expressed genes (*DLD*, *CBR4*, *SURF1*, *MPC2*, *NDUFA4L2*, and *ETFRF1*) were tested using qPCR. Laboratory validation confirmed expression differences detected in RNAseq data for *MT-ND5*, *MT-ND6*, *DLD*, *CBR4*, *SURF1*, *MPC2*, *NDUFA4L2*, and *ETFRF1* (Figure 8).

## 4. Discussion

The main function of the placenta is to provide an appropriate environment for the developing fetus. This function is realized via supplying oxygen and essential nutrients from the maternal circulation, and by extracting carbon dioxide and metabolic waste products from the fetus through the placenta to the maternal circulation [37]. Additionally, the placenta produces hormones and growth factors that are released into the maternal and fetal circulation [38]. Performing these functions is related to a high metabolic rate and oxygen consumption within the placenta. Placental metabolism changes throughout pregnancy and adapts to homoeostatic challenges that come from not only the mother and fetus, but also from the placenta itself. The principal and essential energy substrate for normal placental and fetal metabolism and growth is glucose [39,40]. To meet the high glucose demand of the placenta and developing fetus, its supply is regulated by a compound mechanism that keeps its metabolism relatively constant [1,37]. A part of this mechanism comprises glucose transporters (GLUTs) that mediate facilitated glucose diffusion. GLUTs vary in terms of their substrate specificity, distribution, and regulatory mechanisms [6]. Currently, there are 14 different GLUT proteins that have been characterized in humans, and they are categorized into three main classes based on their sequence homology and functional characteristics. Each GLUT protein has distinct tissue distribution and specific functional characteristics, allowing them to facilitate glucose transport in various physiological contexts. Some GLUTs are constitutively active, while others are insulin-regulated or specific for transporting fructose or urate [8]. Based on current knowledge, the placental glucose transport system is mainly based on SLC2A1 (GLUT1) action. However, it is important to note that other glucose transporters, such as GLUT3 (SLC2A3), GLUT4 (SLC2A4), GLUT8 (SLC2A8), GLUT9 (SLC2A9), GLUT10 (SLC2A10), and GLUT12 (SLC2A12), are also present in the placenta [6,9], which implies they may also be involved in facilitating the uptake of glucose from the maternal bloodstream into placental cells and then supplying the growing fetus. Additionally, such a multitude of different glucose transporters within the placenta may indicate less obvious functions, not necessarily related to direct involvement in glucose uptake. Herein, we report the impact of glucose transporter 8 (SLC2A8) deficiency on the transcriptomic profile of the first-trimester human trophoblast cell line, ACH-3P, and cellular glucose uptake.

Glucose transporter 8 is encoded by the *SLC2A8* gene and has been identified in various tissues, including the placenta. Interestingly, SLC2A8 is a dual-specificity glucose and fructose transporter, and its cellular localization and function differs among tissue types [18,41]. In the blastocyst, SLC2A8 participates in insulin-stimulated cell-membrane hexose transport [19], while in other tissues such as the brain, SLC2A8 is involved exclusively in intracellular hexose transport with no evidence of membrane localization [42]. In the ovine placenta, SLC2A8 is expressed within chorionic epithelium and exhibits increased expression over late gestation [23]. These authors [23] conclude that the decreased placental SLC2A8 concentration observed in an ovine model of placental insufficiency and IUGR may contribute to the reduction in placental glucose transport. Our data indicate that lentiviral-mediated RNA interference resulting in a 79% decrease in the *SLC2A8* mRNA, resulting in an 11% reduction in glucose uptake, which is consistent with their [23] findings. On the other hand, Janzen et al. [43] reported that human IUGR placenta affected by IUGR showed increased SLC2A8 expression and significant differences in SLC2A8 between basal and chorionic plate regions of the placenta [43]. However, Stanirowski et al. [44] examined placental abundance of SLC2A1, SLC2A3, SLC2A8, and SLC2A12 at term, in pregnancies affected by IUGR, SGA (small for gestational age), or macrosomia, and found reduced density of placental SLC2A8 only in SGA pregnancies. In placentas affected by IUGR, SLC2A1 and SLC2A3 were significantly altered, while SLC2A8 remained similar to that in uncompromised pregnancies. No significant differences in any of the four transporters examined were detected in the placentas of macrosomic fetuses [44]. In order to fully address the discrepancies between these studies, the composition of the research cohorts should be considered, as well as the inclusion and exclusion criteria. However, even with that information, not knowing the relative glucose concentrations and placental transfer rate makes it difficult to draw firm conclusions.

Among glucose transporters, SLC2A8 is a class III transporter that is thought to be primarily involved in intracellular transport, rather than glucose uptake across the plasma membrane [18]. Interestingly, in tumors, *SLC2A8* mRNA exists mostly as an untranslated splice variant [45]. It is unclear if this is also the case in normal tissues, or how *SLC2A8* mRNA concentration reflects translated SLC2A8. The possibility that *SLC2A8* mRNA in trophoblast cells is mostly untranslated could explain the discrepancy between the 79% reduction in *SLC2A8* mRNA we obtained and the 11% reduction in glucose uptake. Alternatively, the reduction in glucose uptake may not have directly resulted from SLC2A8-mediated glucose uptake, but indirectly from a reduction in cellular glucose demand for oxidative purposes. The plasticity by which glucose transporters respond to reduced glucose uptake was recently demonstrated by Lynch et al. [14], in which SLC2A3 RNAi resulted in reduced placental glucose uptake in vivo, and a converse increase in SLC2A1 was observed, likely as an attempt to provide adequate glucose to the fetus. The interplay between the various placental glucose transporters, in response to intracellular glucose availability, has not been thoroughly investigated.

Analysis of the *SLC2A8* RNAi transcriptome indicated that diminished *SLC2A8* mRNA mostly affected processes associated with mitochondria function in terms of cellular respiration, oxidative phosphorylation (OXPHOS), and ATP synthesis. This is consistent with previous reports showing that SLC2A8 mostly functions to facilitate hexose transport through intracellular membranes, such as the mitochondrial membrane, endoplasmic reticular membrane, and lysosomal membrane [8,18]. Moreover, the sperm cells of *SLC2A8^−/−^* mice had reduced ATP concentrations and low motility [22], which could result from dysregulated mitochondria function and energy metabolism. Our results may indicate that the reduced glucose uptake due to diminished *SLC2A8* expression results from impaired oxidation of glucose for ATP generation.

Among DEGs that enriched biological processes and metabolic functions, 14 genes were overlapping. Within this group, six genes were downregulated (*ETFRF1*, *NDUFA4L2*, *MPC2*, *SURF1*, *CBR4*, and *DLD*) and eight were upregulated (*MT-ND3*, *MT-ND4*, *MT-ND5*, *MT-ND6*, *MT-CO1*, *MT-ATP6*, *IDH3G*, and *FMO5*). As mitochondria function in the generation of ATP, they are considered the center of metabolism for nearly all eukaryotic cells. However, mitochondria may also participate in a wide range of essential functions related to cellular metabolism, signaling, and programmed cell death [46]. Furthermore, mitochondria function in modulating calcium signaling, which is a universal secondary messenger [47]. Diminished *SLC2A8* expression in trophoblast ACH-3P cells dysregulated *MT-ND3*, *MT-ND4*, *MT-ND5*, and *MT-ND6*, which are the genes encoding subunits of mitochondrial complex I, the first enzyme of the respiratory chain. Complex I is considered the largest and most complex component of the respiratory chain, but at present, the functions of the individual subunits are largely unclear [48]. The primary functions of complex I are to oxidize NADH, generated through the Krebs cycle, and to reduce ubiquinone to ubiquinol. Additionally, complex I is associated with the regulation of reactive oxygen species (ROS), which are important molecules in various signaling pathways, including apoptosis [49]. Disorders of complex I assembly or function affect mitochondria as a whole and may cause major disruption to energy conversion. Severe impairment of mitochondria function is associated with various metabolic disorders, as well as other diseases such as seizures, ataxia, cortical blindness, dystonia, diabetic mellitus, short stature, cardiomyopathy, sensorineural hearing loss, and kidney failure [50]. Therefore, it may be possible that dysregulation of *SLC2A8* expression and glucose uptake leads to disturbed mitochondria function, resulting in pregnancy disorders associated with placental insufficiency and glucose supply below demand.

Among genes that were dysregulated in the currently obtained RNAseq data, several were involved in the formation of cytochrome c oxidase (COX, also known as complex IV), the last enzyme in the respiratory electron transport chain. The COX assembly is a multistep process that involves more than 30 diverse factors, each of which is important for proper COX functioning. SURF1 Cytochrome COxidase Assembly Factor (*SURF1*) encodes one of the assembly proteins involved in the formation of COX. SURF1 disorders are most common and responsible for severe forms of COX deficiency, such as Leigh syndrome [51,52]. Additionally, dysregulation of the Mitochondrially Encoded Cytochrome COxidase I (*MT-CO1*), which encodes one of the central subunits of the COX catalytic core, was detected in the current research. Our current results indicate that in the trophoblast ACH-3P cells, the response to reduced *SLC2A8* involves downregulation of the NDUFA4 Mitochondrial Complex Associated Like 2 (*NDUFA4L2*), a subsequent component of mitochondrial respiratory chain complex IV. NDUFA4L2 is suggested to regulate mitochondrial and lysosomal activities [53]. NDUFA4L2 placental expression is upregulated in response to maternal nutrient restriction in sheep [54]. Additionally, in a sheep model of IUGR, impaired NDUFA4L2 expression was associated with reduction of OXPHOS in skeletal muscle, that in turn was identified as a result of prolonged decrease in the tricarboxylic acid cycle (TCA) and electron transport chain activity [55,56].

Our results indicate that *SLC2A8* deficiency impacted mitochondria functioning multidimensionally, as not only were major mitochondrial enzymes impaired, but carriers of electrons and substrates were also affected. Electron transfer flavoprotein regulatory factor 1 (*ETFRF1*) is associated with the OXPHOS complexes, and interacts with and deflavinates the electron transferring flavoprotein that shuttles electrons to coenzyme Q [57]. Research on energy restriction during late gestation and the muscle and blood transcriptome of beef calves revealed that impaired glucose metabolism may affect genes involved in cellular respiration, including *ETFRF1* [58]. *ETFRF1* was also listed among the genes that were dysregulated in subcutaneous and perirenal adipose tissue of sheep as a resultant of pre- and early postnatal malnutrition [59]. In the current research, *ETFRF1* expression was downregulated as a consequence of the diminished *SLC2A8* expression. Mitochondrially encoded ATP synthase membrane subunit 6 (*MT-ATP6*) contributes to proton-transporting ATP synthase activity. Interestingly, *MT-ATP6* was listed among mitochondrial and glycolysis-regulatory gene expression profiles that are associated with IUGR [60]. Expression of the *MPC2*, the subunit of Mitochondrial Pyruvate Carrier (MPC), was also reduced in the current study. MPC2 creates MPC together with MPC1, and abnormal expression of each of these will lead to MPC dysfunction, which in turn will dysregulate the balance of glycolysis and OXPHOS [61,62].

It should be mentioned that due to the identified dysregulation of genes such as Carbonyl Reductase 4 (CBR4), dihydrolipoamide dehydrogenase (DLD), Flavin Containing Dimethylaniline Monooxygenase 5 (FMO5), and Isocitrate Dehydrogenase (NAD(+)) 3 Non-Catalytic Subunit Gamma (IDH3G), other mitochondrial aspects can also be affected by *SLC2A8* deficiency. Among these, mitochondrial fatty acid synthesis [63,64], as well as disruption of multiple enzyme complexes [65], lipid homeostasis, the uptake and metabolism of glucose, the generation of cytosolic NADPH, the one-carbon metabolism [66], and the TCA cycle, [67], should be listed. An overview of the genes dysregulated due to diminished *SLC2A8* expression supports the conclusion that SLC2A8 is important for proper mitochondria functioning. Impaired expression of the indicated genes, regardless of the direction of change, may cause structural as well as functional mitochondria disruption, manifested by impaired nutrient oxidation and therefore impaired ATP synthesis.

Further functional studies are needed to fully assess SLC2A8 function in the placenta; however, our results regarding reduced glucose uptake and the impact on the transcriptome resulting from *SLC2A8* RNAi, indicate it is reasonable to speculate that *SLC2A8* deficiency could significantly impair placental function. Placental glucose transfer to the fetus depends on the maternal concentration of glucose, the uptake of glucose by the uteroplacental unit, and the maternal/fetal glucose concentration gradient [37,68]. The maternal/fetal concentration gradient, while dependent on the maternal [69] and fetal [37] glucose concentrations, in many ways is also determined by the rate of placental glucose oxidation [16,17]. Collectively, our RNAseq results could infer that a deficiency in placental SLC2A8 would likely impair ATP generation, general metabolic processes, and the redox balance, likely resulting in oxidative stress within the placenta. Placental oxidative stress would have detrimental effects on placental function and fetal development in general, and is considered a factor regulating gene expression and downstream activities such as trophoblast proliferation, invasion, and angiogenesis [70]. Furthermore, the impact of the oxidative stress on placental function is dependent on when during gestation it occurs; however, oxidative stress has been associated with preeclampsia, IUGR, and even pregnancy loss [71,72]. While many factors can influence placental function and placental glucose utilization, our results suggest that SLC2A8, as an intracellular glucose transporter, may play a key role in regulating placental metabolism.

## 5. Conclusions

Using lentiviral-mediated RNA interference in human trophoblast cells, we investigated the impact of specifically reducing the availability of SLC2A8. While SLC2A8 RNAi diminished glucose uptake by these cells, the response was not dramatic and may have been an indirect response. By contrast, there was a major impact on the trophoblast transcriptome, revealing potential impairment in mitochondrial function and oxidative processes. These results align with previous reports in the testes of *Slc2A8^−/−^* mice, resulting in reduced ATP production. As the mammalian placenta utilizes the majority of oxygen and glucose taken up from the maternal circulation, for oxidative processes, our results suggest that SLC2A8 deficiency would likely impair intracellular glucose transport and oxidation, resulting in functional placental insufficiency. Since functional placental insufficiency is a major cause of IUGR, a more thorough understanding of the regulation of placental metabolism is needed, and is highlighted by our results. It should also be kept in mind that placental insufficiency can have lasting effects, impacting the postnatal period and adulthood [73].

## Figures and Tables

**Table 1:** *SLC2A8*-targeting shRNA sequences.

Gene	Accession Number	Oligo Name	Oligonucleotide Sequence (5′ → 3′)
*SLC2A8*	Y17801.1	650 F	CCGGGCTTCTCATGTGCTTCATGCCTTCAAGAGAGGCATGAAGCACATGAGAAGCTTTTTG
		650 R	AATTCAAAAAGCTTCTCATGTGCTTCATGCCTCTCTTGAAGGCATGAAGCACATGAGAAGC
		817 F	CCGGGCATCTACAAGCCCTTCATCATTCAAGAGATGATGAAGGGCTTGTAGATGCTTTTTG
		817 R	AATTCAAAAAGCATCTACAAGCCCTTCATCATCTCTTGAATGATGAAGGGCTTGTAGATGC
		1452 F	CCGGGGAAAGACTCTGGAACAAATCTTCAAGAGAGATTTGTTCCAGAGTCTTTCCTTTTTG
		1452 R	AATTCAAAAAGGAAAGACTCTGGAACAAATCTCTCTTGAAGATTTGTTCCAGAGTCTTTCC
		1863 F	CCGGGCCTTATCGGGAAGGAAATTTTTCAAGAGAAAATTTCCTTCCCGATAAGGCTTTTTG
		1863 R	AATTCAAAAAGCCTTATCGGGAAGGAAATTTTCTCTTGAAAAATTTCCTTCCCGATAAGGC

**Table 2:** Primers used for qPCR of *SLC2A8* mRNA.

Gene	Accession Number	Fwd (5′ → 3′)	Rev (5′ → 3′)	Amplicon Size (bp)
*SLC2A8*	Y17801.1	ATGTGCTTCATGCCCGAGACC	TGGATGACACCCACGACGA	311
*RPS15*	NM_001018	TTCCGCAAGTTCACCTACC	CGGGCCGGGCATGCTTTACG	361

**Table 3:** Sequencing, quality control, and mapping metrics for control and *SLC2A8*-deficient ACH-3P cells.

	Control ACH-3P Cells				*SLC2A8*-Deficient ACH-3P Cells			
	1	2	3	4	1	2	3	4
**Row reads**	101,985,422	119,212,310	117,319,316	122,204,792	122,233,048	138,670,320	127,242,242	1.01 × 10^8^
**Mapped reads**	58,421,944	67,375,772	65,818,390	66,997,252	67,896,148	74,225,838	69,811,368	54,391,282
**Uniquely mapped reads**	56,626,194	65,017,284	63,508,560	64,650,006	65,217,452	71,508,530	67,218,322	52,358,886
**Multi-mapped reads**	1,789,424	2,348,664	2,301,056	2,338,934	2,668,114	2,706,906	2,584,922	2,024,950
**Too many loci**	6326	9824	8774	8312	10,582	10,402	8124	7446
**Expressed transcripts**	58,147	60,405	59,872	60,476	60,606	62,106	61,764	58,481
**Expressed genes**	24,084	25,154	24,971	24,909	24,910	25,194	24,965	24,357

## Data Availability

The sequencing data from this study have been submitted to the European Nucleotide Archive under accession no. PRJEB71894 (https://www.ebi.ac.uk/ena, 16 January 2024).
